# Papillary Glioneuronal Tumor Located in the Subcortical White Matter With a Purely Solid Pattern: A Case Report

**DOI:** 10.7759/cureus.82340

**Published:** 2025-04-15

**Authors:** Masayuki Hirata, Osamu Togao, Koji Yamashita, Kazufumi Kikuchi, Masaoki Kusunoki, Fumiya Narutomi, Daisuke Kuga, Yutaka Fujioka, Koji Yoshimoto, Kousei Ishigami

**Affiliations:** 1 Department of Clinical Radiology, Graduate School of Medical Sciences, Kyushu University, Fukuoka, JPN; 2 Department of Molecular Imaging and Diagnosis, Graduate School of Medical Sciences, Kyushu University, Fukuoka, JPN; 3 Department of Anatomic Pathology, Pathological Sciences, Graduate School of Medical Sciences, Kyushu University, Fukuoka, JPN; 4 Department of Neurosurgery, Graduate School of Medical Sciences, Kyushu University, Fukuoka, JPN

**Keywords:** brain tumor, hemosiderin deposition, neuro mri, papillary glioneuronal tumor, pgnt

## Abstract

Papillary glioneuronal tumor (PGNT) is a rare brain tumor classified as a CNS WHO grade 1 tumor. It commonly occurs in the periventricular white matter and typically presents as a well-circumscribed mass composed of both cystic and solid components. In this report, we present a rare case of PGNT arising in the subcortical white matter and consisting entirely of solid components. Notably, a low-signal intensity rim was observed at the tumor margins on susceptibility-weighted imaging, a feature previously reported in PGNT. This finding may have diagnostic significance, as it could help differentiate PGNT from other tumors with overlapping imaging features, such as ganglioglioma, pleomorphic xanthoastrocytoma, and brain metastasis.

## Introduction

Papillary glioneuronal tumor (PGNT) is a rare type of brain tumor first reported by Komori et al. in 1998 and is classified as a CNS WHO grade 1 tumor in the 2021 classification of tumors of the CNS [1,2]. More than 120 cases of PGNT have been reported to date in the literature [3-8]. It has been reported that the median age of onset is 23 years, with a higher incidence in younger populations [4]. Symptoms include headache, nausea, vomiting, and seizures [4]. The primary treatment is complete tumor resection, and it has a favorable prognosis; the 1.5-year progression-free survival and overall survival rates were 86% and 98%, respectively [4].

It has been reported that most PGNTs are located in the cerebral white matter, with a predilection for periventricular regions, and they typically consist of cystic and solid components [3-5]. Here, we report an atypical case of PGNT that originated in the subcortical white matter and was purely composed of solid components. Preoperative radiological differentiation of PGNT from tumors with similar imaging features, such as pleomorphic xanthoastrocytoma (PXA), ganglioglioma, and brain metastasis, is clinically important. While PGNT, PXA, and ganglioglioma are low-grade tumors, brain metastasis is malignant and requires systemic evaluation. The PXA may undergo anaplastic transformation, and gangliogliomas are often associated with epilepsy, influencing surgical strategy. Accurate diagnosis supports appropriate surgical planning, patient counseling, and postoperative management.

## Case presentation

The patient is a 33-year-old male who presented with a chief complaint of right facial spasm. He had been experiencing right facial spasms for one month. He consulted a previous physician and underwent an MRI scan, which revealed an intracranial mass lesion. Based on the findings, he was diagnosed with symptomatic focal epilepsy. The patient was subsequently referred to our hospital for further examination and treatment. His past medical history and family history were unremarkable. Neurological examination revealed right facial spasm, with no other significant findings. 

**Figure 1:**
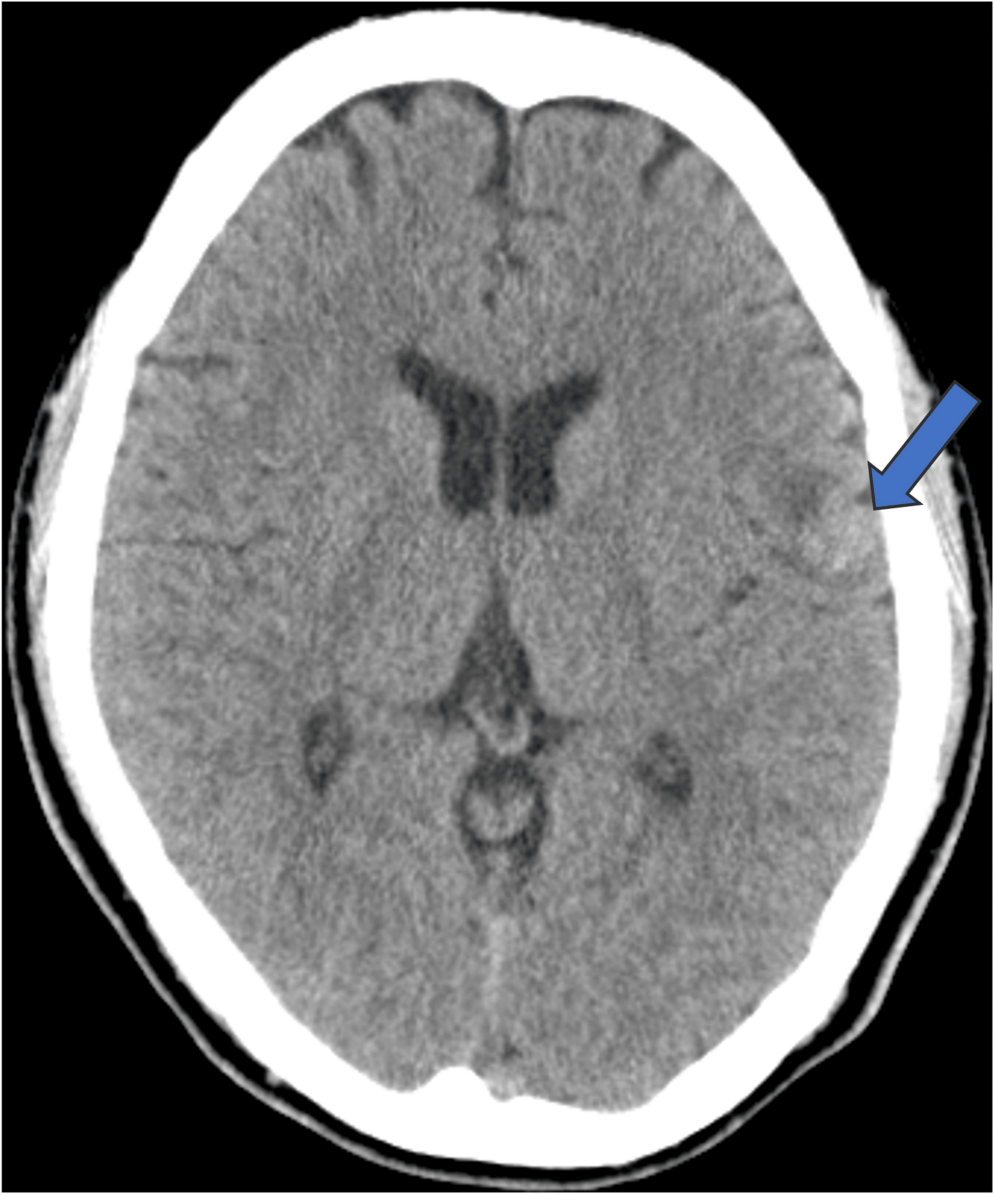
Plain CT shows the mass located in the left frontoparietal opercular subcortical white matter extending into the cortex (arrow). The mass shows isoattenuation compared to the normal gray matter, with surrounding white matter displaying low-attenuation areas suggestive of edematous changes. No obvious calcification or hemorrhage is observed.

On plain CT, the mass was observed in the left frontoparietal opercular subcortical white matter extending into the cortex. The mass showed isoattenuation compared to the normal gray matter, and no obvious calcification or hemorrhage was observed (Figure 1).

**Figure 2:**
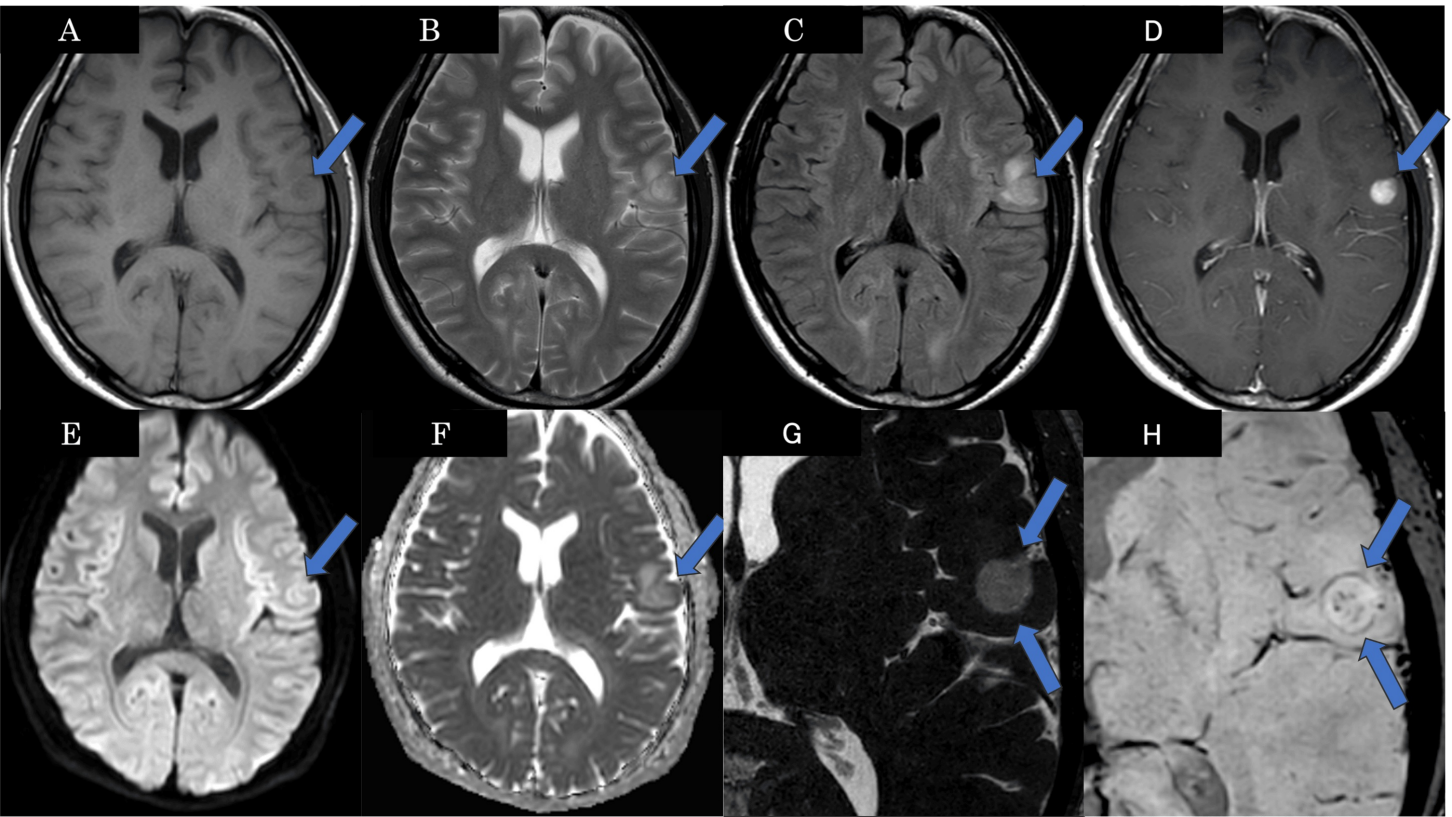
Brain MRI of the patient The well-circumscribed mass (arrows) appears hypointense on T1WI (A), hyperintense on T2WI (B) and FLAIR (C), and demonstrates solid enhancement on contrast-enhanced T1WI (D). There is no signal elevation on DWI (E), and the apparent diffusion coefficient (ADC) map (F) shows a mean ADC value of 1.10×10-3 mm²/sec. Constructive interference in steady state (CISS) imaging (G) reveals no cystic component within the tumor. The SWI (H) demonstrated rim-like low signal intensity at the periphery and punctate low signal areas internally, indicative of hemosiderin deposition. T1WI: T1-weighted image, T2WI: T2-weighted image, FLAIR: Fluid-attenuated inversion recovery, DWI: Diffusion-weighted image, SWI: Susceptibility-weighted imaging

On brain MRI, the mass was well-circumscribed, appearing hypointense on T1-weighted images (T1WI), hyperintense on T2-weighted images (T2WI), and fluid-attenuated inversion recovery (FLAIR), and demonstrated purely solid enhancement on contrast-enhanced T1WI. There was no restricted diffusion on diffusion-weighted imaging (DWI). Susceptibility-weighted imaging (SWI) demonstrated a rim-like low-signal intensity at the periphery, suggestive of peripheral hemosiderin deposition (Figure 2).

**Figure 3:**
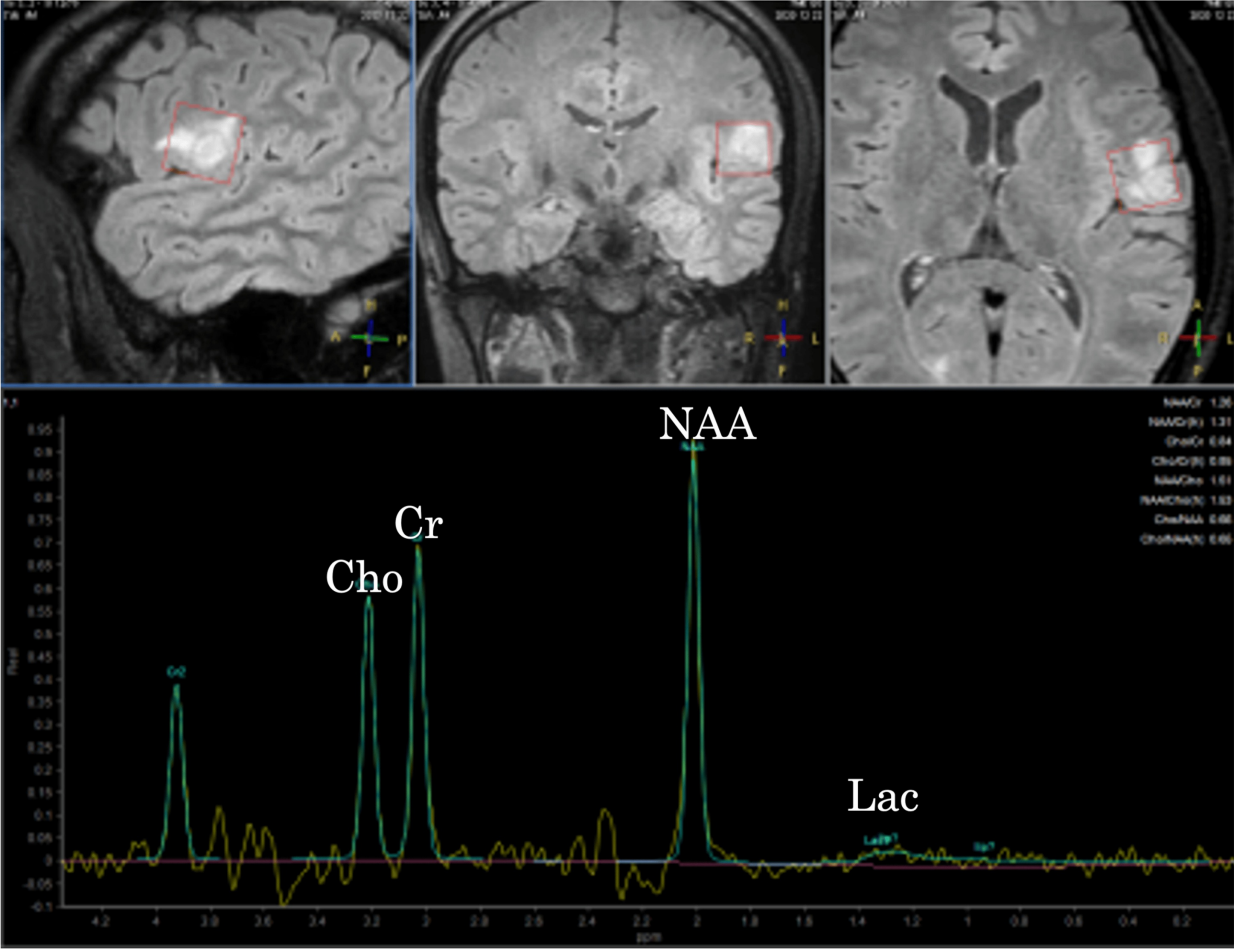
The MRS imaging The ^1^H-magnetic resonance spectroscopy (^1^H-MRS) with an echo time (TE) of 97 ms shows neither an elevated choline-to-creatine ratio nor a lactate peak; findings consistent with a benign tumor.

**Figure 4:**
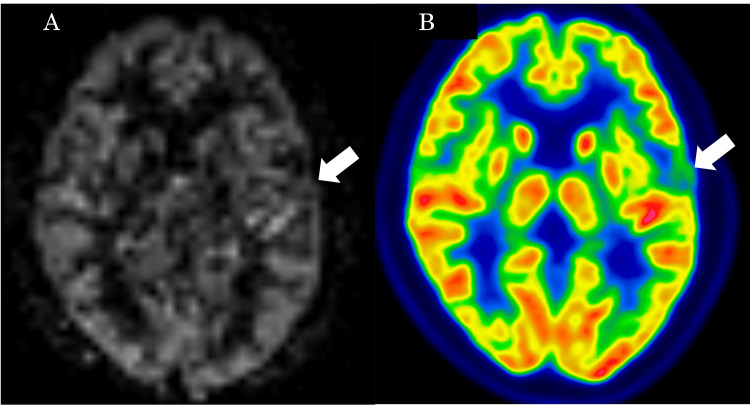
The ASL and FDG-PET scan Pseudo-continuous ASL (A) shows no obvious hyperperfusion within the tumor, and FDG-PET (B) shows no abnormal FDG uptake with a maximum standardized uptake value (SUV) max of 4.73 in the tumor (arrows). ASL: Arterial spin labeling, FDG-PET: Fluorodeoxyglucose positron emission tomography

The ^1^H-magnetic resonance spectroscopy (^1^H-MRS) did not show an elevated choline/creatine ratio or lactate peak (Figure 3). Pseudo-continuous arterial spin labeling (ASL) showed no obvious hyperperfusion within the tumor. Fluorodeoxyglucose positron emission tomography (FDG-PET) revealed no abnormal FDG uptake in the tumor (Figure 4). Based on these imaging findings, PXA and ganglioglioma were considered as top differential diagnoses. A gross total resection was performed.

**Figure 5:**
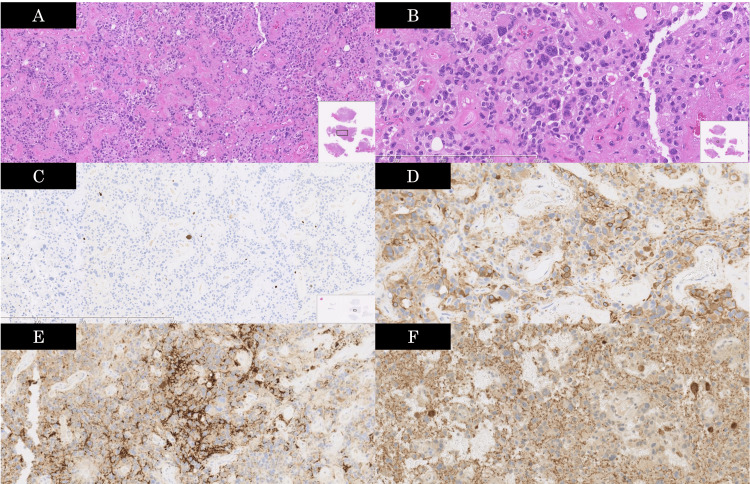
Pathological images A: Tumor cells arranged in a pseudo-papillary pattern around hyalinized blood vessels 9original magnification ×80; H&E stain); B: Proliferation of round to polygonal hyperchromatic tumor cells, with some areas showing highly atypical cells and multinucleated giant cells (original magnification ×200; H&E stain); C: MIB-1 index of less than 5% (original magnification ×80; Ki-67 stain); D: GFAP-positive cells arranged in a pseudo-papillary pattern around blood vessels (original magnification ×200; GFAP stain); E: Interpapillary neuronal elements positive staining for synaptophysin (original magnification ×200; synaptophysin stain); F: Interpapillary neuronal elements positive staining for NeuN (original magnification ×200; NeuN stain). The histopathological diagnosis is papillary glioneuronal tumor. H&E: Hematoxylin and eosin, GFAP: Glial fibrillary acidic protein, NeuN: Neuronal nuclei

On histopathology, hematoxylin-eosin staining revealed tumor cells arranged in a pseudo-papillary pattern around hyalinized blood vessels. The Ki-67 staining showed an MIB-1 antibody-based Ki-67 labeling index of less than 5%. Glial fibrillary acidic protein (GFAP), neuronal nuclei (NeuN), and synaptophysin were all positive (Figure 5). Isocitrate dehydrogenase (IDH) was wildtype. The final histopathological diagnosis was papillary glioneuronal tumor. Four years postoperatively, there has been no evident recurrence, and the patient remains stable.

## Discussion

We report a case of PGNT that developed in the cerebral subcortical white matter. Histopathologically, it is characterized by the presence of GFAP-positive small cuboidal cells lining hyalinized vascular pseudopapillae, along with synaptophysin and/or NeuN-positive interpapillary neuronal elements [1,3,5,9].

A PGNT is predominantly supratentorial, with a predilection for periventricular regions, and rarely occurs in the subcortical white matter. It typically presents as a well-defined contrast-enhancing cystic-solid mass, but it can also be pure solid or cystic. It was previously reported that a purely solid pattern was rare, accounting for only 7% of cases [4]. Solid components of the tumor are iso/hypointense on T1WI and hyperintense on T2WI/FLAIR, which is consistent with the present case [6].

In this case, the tumor's location in the subcortical white matter and its purely solid nature made it atypical for PGNT, complicating preoperative differentiation from PXA or ganglioglioma. It was reported that PGNTs show hypointense or isointense on DWI and did not show restricted diffusion [3,5]. This is consistent with the present case. The ^1^H-MRS and perfusion analysis are described in very few studies; however, increased choline creatinine ratio and hyperperfusion tend to be seen in the recurrent PGNT [3,10]. Similarly, reports on FDG-PET findings for this tumor are also limited. Shinno et al. reported the decreased FDG uptake in the tumor compared to normal brain parenchyma [11]. The findings of the present case in ^1^H-MRS, ASL, and FDG-PET are consistent with those of a benign tumor.

Several reports have noted low signal areas at the tumor margins on SWI in PGNTs, suggesting hemosiderin deposition [3,12]. This case also showed a low signal rim at the margins on SWI. On histopathology, hemosiderin deposits are often found at the margins of PGNT as described in the WHO CNS 5th edition [2]. The low-signal rim on SWI, as observed in this case, may support a preoperative diagnosis of PGNT, as it is not a characteristic finding in tumors with similar imaging appearances, such as PXA, ganglioglioma, or brain metastasis. Recognition of this imaging feature can aid in achieving a more accurate preoperative diagnosis and in optimizing surgical planning.

## Conclusions

We reported an atypical case of PGNT that originated in the cerebral subcortical white matter and presented with a purely solid pattern. When exhibiting atypical features, preoperative diagnosis of PGNT can be challenging. The presence of a low signal intensity rim at the tumor margins on SWI may be a useful imaging finding in the diagnosis of PGNT, even in atypical cases.
